# Enhancing the Initial Whiteness and Long-Term Thermal Stability of Polyvinyl Chloride by Utilizing Layered Double Hydroxides with Low Surface Basicity

**DOI:** 10.3390/polym15041043

**Published:** 2023-02-19

**Authors:** Guanhua Shen, Yanhua Zhao, Mingxin Ma, Yongli Wang, Xiangying Hao, Guodong Yuan

**Affiliations:** 1 Guangdong Provincial Key Laboratory of Environmental Health and Land Resource, College of Environmental and Chemical Engineering, Zhaoqing University, Zhaoqing 526061, China; 2 College of Chemistry & Environmental Science, Inner Mongolia Normal University, Hohhot 010022, China

**Keywords:** layered double hydroxides, thermal stabilizer, thermal stability, poly(vinyl chloride) composite, surface basicity

## Abstract

This study investigated the impact of surface basicity on the performance of layered double hydroxides (LDHs) as heat stabilizers for polyvinyl chloride (PVC). LDHs with varying surface basicity were synthesized and characterized using XRD, SEM, BET, and CO_2_-TPD. The LDHs were then combined with zinc stearate and dibenzoylmethane to create an environmentally friendly heat stabilizer and added to PVC. The resulting PVC composites were evaluated for thermal stability using the oven-aging method. The results showed that a lower Mg/Al molar ratio (2.0) improved the initial whiteness and long-term thermal stability of PVC composites compared to higher ratios (2.5, 3.0, and 3.5). Replacing Mg with Zn in the LDHs had a similar effect to that of reducing the Mg/Al ratio. Crosslinking the laminae of LDHs with 5% silane coupling agent KH-560 reduced the surface basicity of LDHs by 79%, increasing the chromaticity index, b*, and thermal stability time of PVC composites by 48% and 14%, respectively. A descriptive relationship was established between the structure and surface basicity of LDHs and the initial whiteness and long-term thermal stability of PVC composites.

## 1. Introduction

Polyvinyl chloride (PVC) is a versatile polymer with high strength, corrosion resistance, durability, good insulation properties, and low-cost. These properties have led to it wide use in a variety of applications, including building materials, packaging, furniture, decorative items, electronics, automotive parts, and medical equipment [1,2]. Despite its advantages, PVC is known for its poor thermal stability, which can cause it to decompose and release hydrogen chloride gas (HCl) at temperatures as low as 90 °C, leading to a decrease in its mechanical and electrical properties. To address this issue, thermal stabilizers are added to PVC during processing to prevent or slow down its decomposition [3,4,5,6,7]. However, the traditional thermal stabilizers, such as lead/cadmium salts and organotin, pose health and environmental hazards, leading to regulations such as EU 76/769/EEC, WEEE, and RoHS that require the use of non-toxic, environmentally friendly, and efficient thermal stabilizers [8]. Thermal stabilizers of β-diketone compounds, zinc stearate, and LDHs show great potential as practical alternatives [4,9,10,11,12,13,14].

Layered double hydroxides (LDHs) are a type of anionic compound with a formula of [$M_{1-x}^{2+}M_{x}^{3+}{\left(\mathrm{OH}\right)}_{2}$]^x+^ [${\left(A^{n-}\right)}_{\frac{x}{n}}$▪mH_2_O]^x−^, where M^2+^ is a divalent metal ion, M^3+^ is a trivalent metal ion, and A^n−^ is an interlayer anion. The molecular formula for magnesium aluminum carbonate hydrotalcite, a typical LDH, is Mg_6_Al_2_(OH)_16_CO_3_·4H_2_O [14]. Due to their basicity and ion exchange abilities, LDHs can absorb the HCl released during PVC decomposition, reducing the impact of HCl on the decomposition process and thereby enhancing PVC’s thermal stability [14,15]. By changing the metal elements in the laminae, the interlayer anions, the surface properties, and the grain size and size distribution of LDHs, the thermal stability of PVC can be improved [16,17,18,19,20,21,22,23,24,25,26].

The role of LDHs in enhancing the whiteness of PVC is believed to stem from the positive charge of LDH platelets interacting with the electron clouds of vinyl chloride and tertiary chlorine atoms in PVC chains, which weakens the activity of chlorine atoms and restricts PVC dehydrochlorination. The long-term thermal stability of PVC is related to HCl absorption by LDHs, either through ion exchange between interlayer anions and HCl or platelet hydroxides absorbing HCl. The exact mechanism is still uncertain. However, Lin et al. [27] found a positive correlation between the amount of CO_3_^2−^ in LDHs and the long-term thermal stability of PVC, and a negative correlation between the amount of -OH and the total HCl absorption capacity, leading to the conclusion that ion exchange between interlayer anions and HCl is the critical factor in enhancing PVC thermal stability. The interlayer anions in LDHs must overcome the reaction between surface hydroxides and HCl to facilitate this exchange.

The basicity of LDHs plays a crucial role in adsorbing HCl and reducing the catalytic decomposition of PVC. On the other hand, alkaline substances can also directly attack PVC molecular chains and promote HCl release, thus generating a conjugated polyene sequence and making the PVC molecular chain less stable [28]. Solid-state nuclear magnetic resonance research showed that with changes in the molar ratio of the metal elements in the layers of LDHs and the types of interlayer anions, the hydroxyl environment of the LDHs also changed [29,30]. The latter will inevitably lead to changes in the basicity of LDHs. Zhang et al. [24] have confirmed that changing the type of interlayer anions in LDHs leads to a significant change in the surface basicity of LDHs, which demonstrates that the surface basicity of LDHs cannot be ignored when discussing the impact of LDHs on the thermal stability of PVC. To our best knowledge, little has been reported on the effect of the basicity of LDHs on PVC properties.

The relationship between the structure and properties of LDHs and the initial whiteness and long-term thermal stability of PVC is not well understood. This study aimed to fill that gap by investigating the impact of various factors, such as metal element type and proportion, hydroxyl type, and crosslinking degree of the laminate, on the surface basicity of LDHs. The goal was to uncover the relationship between the surface basicity of LDHs and the thermal stability of PVC, with the ultimate objective of developing high-performance LDHs for use as PVC heat stabilizers.

## 2. Materials and Methods

### 2.1. Materials

PVC (SG–5) was obtained from Xinjiang Tianye (Group) Co., Ltd. (Shihezi, China). Dioctyl terephthalate (DOTP, 99%) was from Nantong Runfeng Petrochemical Co., Ltd. (Nantong, China). Calcium carbonate (98%) was purchased from Guangxi Xin Gai Mining Co., Ltd. (Guilin, China). Thermal stabilizer (TS-Zn) with zinc stearate and dibenzoylmethane as the key components was from Shanghai Haoyuan Environmental Protection Material Co., Ltd. (Shanghai, China). Mg(NO_3_)_2_·6H_2_O, Al(NO_3_)_3_·9H_2_O, Ca(NO_3_)_2_·4H_2_O, Zn(NO_3_)_2_·6H_2_O, NaOH, and NaHCO_3_, all analytical grade, were purchased from Guangzhou Chemical Reagent Factory (Guangzhou, China). Silane coupling agent (KH-560, 98%) was provided by Nanjing QiyuChem (Nanjing, China).

### 2.2. Preparation of LDHs

Preparation of MgAl-CO_3_ LDHs: First, 17.20 g Mg(NO_3_)_2_·6H_2_O and 12.58 g Al(NO_3_)_3_·9H_2_O were added into 80 mL distilled water to form solution A to give n(M^2+^/M^3+^) = 2, n(OH^−^)/(2n(M^2+^) + 3n(M^3+^)) = 1.0, and n(HCO_3_^2−^)/n(M^3+^) = 0.5. Then, 9.39 g NaOH was dissolved in 60 mL of distilled water to form solution B, and 1.40 g NaHCO_3_ was dissolved in 20 mL distilled water to form solution C. Furthermore, solution A was placed into a 250 mL flask, and solution B was added drop by drop to the flask under stirring at 80 °C until finished and then continuously stirred for 10 min. Then, solution C was added drop by drop to the flask. The obtained slurry was crystallized at 80 °C for 12 h, followed by suction filtration, washing, drying, and grinding to obtain Mg/Al hydrotalcite (labeled as MgAl2.0). The above procedures were repeated for n(M^2+^/M^3+^) = 2.5, 3.0, and 3.5 to produce Mg/Al hydrotalcite with different Mg/Al molar ratios (labeled as MgAl2.5, MgAl3.0, and MgAl3.5, respectively).

Preparation of CaAl-CO_3_ and ZnAl-CO_3_ LDHs: Similar to the synthesis of MgAl-CO_3_ LDHs, Ca(NO_3_)_2_·4H_2_O or Zn(NO_3_)_2_·6H_2_O was used to replace Mg(NO_3_)_2_·6H_2_O, with n(OH^−^)/(2n(M^2+^) + 3n(M^3+^)) = 1.0, n(HCO_3_^2−^)/n(M^3+^) = 0.5, to produce Ca-Al LDHs and Zn-Al LDHs (labeled as CaAl2.0 and ZnAl2.0, respectively).

Preparation of CaMgAl-CO_3_ and ZnMgAl-CO_3_ LDHs: Similar to the preparation of MgAl-CO_3_ LDHs, Ca(NO_3_)_2_·4H_2_O or Zn(NO_3_)_2_·6H_2_O was used to partially replace Mg(NO_3_)_2_·6H_2_O, with n(Mg^2+^/Ca^2+^) = 1 or n(Mg^2+^/Zn^2+^) = 1, n(M^2+^/M^3+^) = 2, n(OH^−^)/(2n(M^2+^) + 3n(M^3+^)) = 1.0, and n(HCO_3_^2−^)/n(M^3+^) = 0.5, to produce Ca-Mg-Al LDHs and Zn-Mg-Al LDHs (labeled as CaMgAl11 and ZnMgAl11, respectively).

Preparation of cross-linked Mg-Al LDHs: The method of preparing Mg-Al-CO_3_ LDHs was followed. Specifically, 0.32 g (5 wt % Mg-Al LDHs) of silane coupling agent KH-560 was added to solution A, then solutions B and C were added drop by drop, and the slurry was stirred at 160 °C for 12 h to obtain cross-linked Mg-Al LDHs, labeled as CL-MgAl2.0.

### 2.3. Characterization of LDHs by XRD, CO_2_-TPD, SEM, BET, and Particle Size

The obtained LDHs were analyzed by an X-ray diffractometer (D8 ADVANCE, Bruker, Billerica, MA, USA), with the following characteristics: Cu-Kα radiation, 40 kV voltage, 40 mA current, a scanning range of 5–70°, and a scanning speed of 10°/min.

The surface basicity of LDHs was determined by an automatic chemical adsorption instrument (ChemBET Pulsar TPR/TPD, Quantachrome Instruments, Boynton Beach, FL, USA) by weighing 60 ± 2 mg of samples, purging with helium at 150 °C for 30 min, lowering the temperature to 60 °C, allowing the adsorption of CO_2_ gas for 30 min, and then purging the CO_2_ on the surface of the sample by helium gas. Desorption was carried out by using helium as the carrier gas at a flow rate is 80 mL/min and a heating rate of 10 °C/min.

The morphology of the LDHs was observed using a field emission scanning electron microscope (ZEISS SUPRA 55, ZEISS, Jena, Germany) with a voltage of 5 kV.

An automatic multifunctional gas adsorption instrument (ASAP 2020, Micromeritics Instrument Corporation, Norcross, GA, USA) was used to determine the specific surface area of LDHs. The samples were vacuum-dried at 150 °C for 2 h, and a nitrogen adsorption isotherm at 77 K was used to calculate the BET-specific surface area.

The particle size and size distribution of the LDHs were measured using the HORIBA LA-960 laser particle size analyzer with air as the dispersant, and the pressure was set at 0.4 MPa.

### 2.4. Preparation of PVC Composites

**Figure 1:**
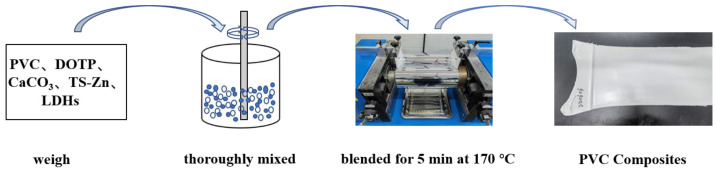
A schematic diagram of the preparation of PVC composites.

As shown in Figure 1, 100.0 g of PVC, 50.0 g of DOTP, 30.0 g of CaCO_3_, and 3.0 g of Zn-based thermal stabilizer composed of 1.0 g of TS-Zn and 2.0 g of LDHs were thoroughly mixed and blended in a double-roller mixer that was heated to 170 °C. The resulting PVC composites were compression molded to a 0.8-mm thick film.

### 2.5. Thermal Stability Tests of PVC Composites

Oven-aging tests on the PVC composite samples were performed at 200 °C following the Chinese National Standard Testing Method GB/T 9349–2002 [31].

Color measurements of the PVC composites were preformed using a high-quality portable computer colorimeter (NH310, Shenzhen Threenh Technology Co., Ltd., Shenzhen, China) with the following specifications: light source D65, test aperture Φ8 mm, and display mode CIE L*a*b*C*H*. The lightness index, L*, indicates black and white between 0 and 100. The chromaticity index, a*, represents the red-green axis, with positive values indicating red and negative values indicating green. The chromaticity index, b*, represents the yellow-blue axis, with positive values indicating yellow and negative values indicating blue. A yellowish shade means the discoloration of the PVC sample during the initial heating stage, and the change in b* can be used to characterize its whiteness. The greater the b* value, the darker the yellow shade.

The morphology of the PVC composites before and after aging was observed using a ZEISS Sigma 300 (ZEISS, Jena, Germany), and the attenuated total reflection infrared spectra of the PVC composites before and after aging were measured using a Shimadzu IRTracer-100 (Shimadzu Corporation, Kyoto, Japan).

## 3. Results and Discussion

### 3.1. Effect of Metal Species in the Laminae on the Surface Basicity of LDHs and the Thermal Stability of PVC Composites

**Figure 2:**
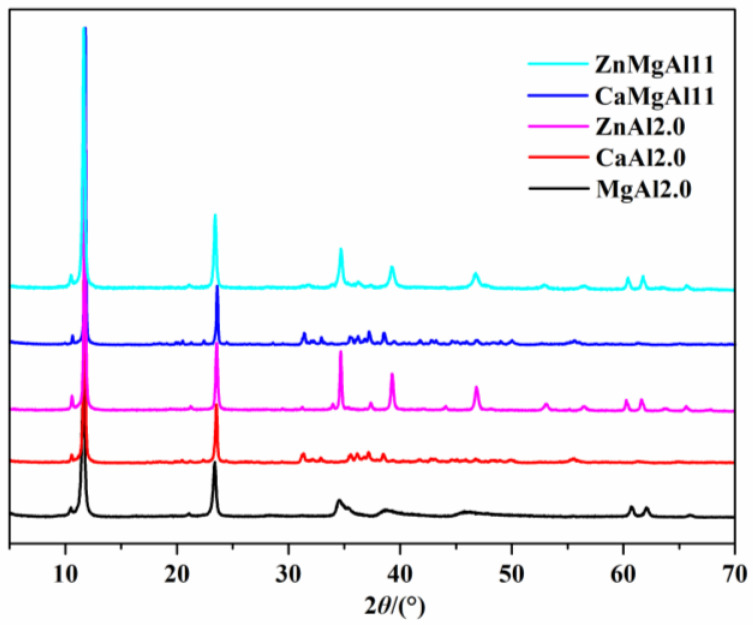
The XRD patterns of LDHs with various metal elements in the layers.

As demonstrated in Figure 2, when the metal elements in the layers were Mg and Al, strong diffraction peaks were observed at 2θ angles of 11.61°, 23.39°, 34.50°, 38.69°, 60.70°, and 62.05°, which correspond with those of PDF#89-0460, indicating the successful synthesis of an Mg-Al hydrotalcite. After the Mg in the hydrotalcite layers was fully substituted with Ca or Zn, the d(003) diffraction peak shifted to larger angles of 11.69° and 11.71°, respectively, which is consistent with PDF#87-0493 and PDF#48-1023, signifying the preparation of Ca-Al or Zn-Al LDHs. When the ratios of n(Mg)/n(Zn)/n(Al) were 1:1:1, the resulting product displayed characteristic diffraction peaks of typical LDHs, and the d(003) diffraction peak was between that of Mg-Al and Zn-Al LDHs. Similarly, for the ratios of n(Mg)/n(Ca)/n(Al) = 1:1:1, the d(003) diffraction peak of the product was located between those of Mg-Al and Ca-Al LDHs. XRD patterns confirmed the formation of Zn-Mg-Al and Ca-Mg-Al LDHs [32].

The layer charge density and the size of the interlayer anions play a role in determining the interlayer spacing in LDHs. A higher layer positive charge density results in a larger interlayer spacing. This relationship can be observed through the d(003) diffraction peak position in LDHs, with the order of MgAl2.0 > CaMgAl11 > ZnMgAl11 > CaAl2.0 > ZnAl2.0.

**Figure 3:**
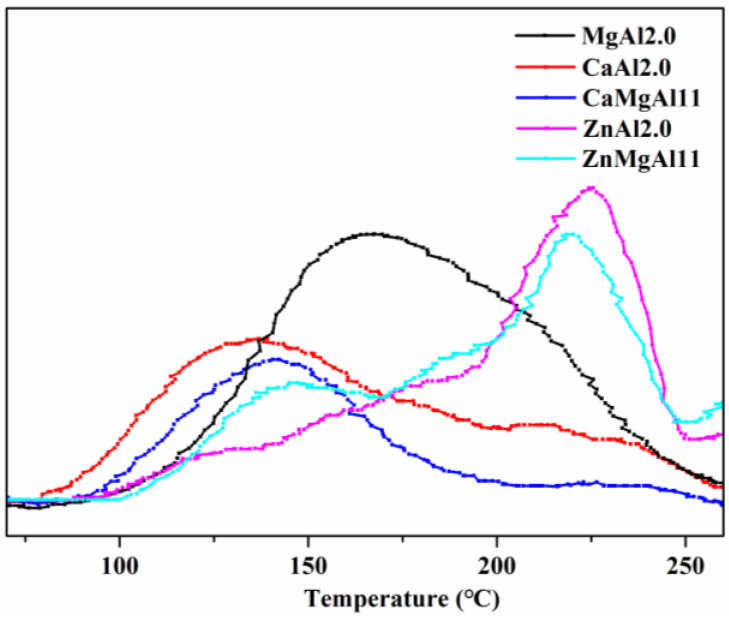
CO_2_-TPD profiles of LDHs with varying metal elements in the laminae.

Temperature-programmed desorption (TPD) is a crucial technique used to study the basicity of material surfaces. A higher temperature for the CO_2_ desorption peak suggests stronger basicity, and a larger area of the desorption peak indicates a greater amount of basicity [33]. Weak basicity (<200 °C) of LDHs was mainly attributed to the hydroxyl group, while medium to strong basicity (200–300 °C) was due to M-O (where M is a metal element) [24]. The CO_2_-TPD diagrams (Figure 3) and the derived desorption peak temperature and area (Table 1) indicate that Mg-Al hydrotalcite (MgAl2.0) had a desorption peak temperature of 165 °C. This decreased to 141 °C and 134 °C when Mg was partially replaced (CaMgAl11) or fully replaced (CaAl2.0) by Ca, respectively. On the other hand, partial or full replacement of Mg by Zn (ZnMgAl11 and ZnAl2.0) increased the desorption peak temperature to 218 °C and 225 °C, respectively. This is probably because zinc atoms have empty orbitals in the outer layer, and Zn-O has stronger polarity than Mg-O and Al-O. Therefore, the basic strength of LDHs decreased in the order of ZnAl2.0 > ZnMgAl11 > MgAl2.0 > CaMgAl11 > CaAl2.0, which is opposite to the basic strength order of metal hydroxides Ca(OH)_2_ > Mg(OH)_2_ > Zn(OH)_2_. In other words, metal species in the laminae greatly affect the surface basic strength of LDHs, where the higher the basic strength of metal hydroxide, the weaker the surface basic strength of LDHs. The desorption peak area follows the order of MgAl2.0 (1188) > ZnAl2.0 (982) > ZnMgAl11 (971) > CaAl2.0 (609) > CaMgAl11(458). This order is different from that of basicity, suggesting that factors other than metal species also impact the surface basicity of LDHs.

**Figure 4:**
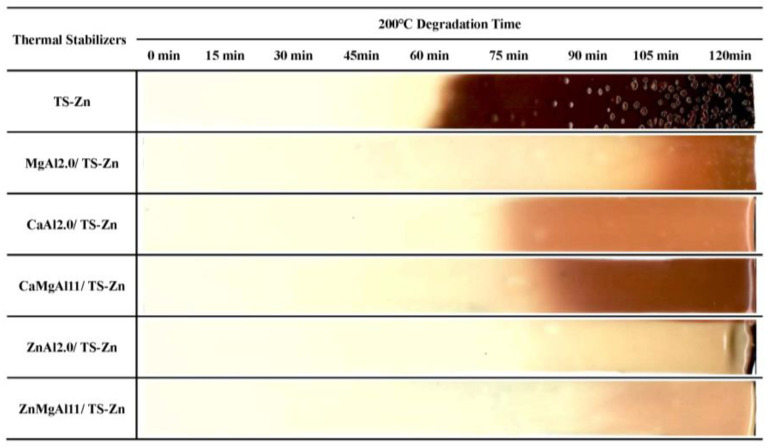
Metal species in laminae of LDHs affect the thermal stability of PVC composites in an oven-aging test.

The oven-aging method was employed to evaluate the impact of LDHs on the thermal stability of PVC composites (as shown in Figure 4). This method involves the thermal decomposition of PVC, which results in the release of HCl from the PVC molecular chain and the formation of a conjugated polyene sequence (CPS). As the CPS increases, the color of PVC changes from white to light yellow, yellow, orange, brown, and eventually black, as reported in [10].

In the early stage (from 0 min to the color turning yellow) of PVC aging at 200 °C, the chromaticity index, b*, can indicate the degree of yellowing. The smaller b* is, the further the color from yellow [34]. For PVC composites with 1.0 g of stabilizer (TS–Zn) only, at an aging time of 0 min, the chromaticity index b* was 4.08, indicating an excellent initial whiteness of PVC. Adding CaAl2.0, CaMgAl11, ZnMgAl11, and ZnAl2.0 to PVC changed the b* to 7.14, 6.73, 6.53, and 6.38, respectively. In other words, the PVC color became lighter in the order of thermal stabilizers CaAl2.0, CaMgAl11, ZnMgAl11, and ZnAl2.0. Thus, the ability of LDHs to improve the initial whiteness decreased in the order of ZnAl2.0 > ZnMgAl11 > CaMgAl11 > CaAl2.0. This order contradicts the one of positive layer charge density of LDHs (MgAl2.0 > CaMgAl11 > ZnMgAl11 > CaAl2.0 > ZnAl2.0), indicating that the positive layer charge density of LDHs is not fully correlated with the initial whiteness of PVC. In contrast, the ability of LDHs to improve the initial whiteness agrees with the decreasing order of basic strength ZnAl2.0 > ZnMgAl11 > CaMgAl11 > CaAl2.0. Based on these findings, it can be concluded that increasing the basic strength of LDHs leads to an improved initial whiteness of PVC composites.

At 60 min of aging, the PVC with TS-Zn stabilizer turned black, a phenomenon known as “zinc burning.” This was due to the absorption of HCl released from PVC decomposition by zinc stearate in TS-Zn, leading to the formation of ZnCl2, which catalytically accelerated PVC decomposition [12]. PVC composites with MgAl2.0/TS-Zn had an initial chromaticity index b* of 7.62 and, although its initial whiteness was not as good as PVC stabilized with TS-Zn only, its color did not turn yellow until 60 min of aging and did not turn brown until 105 min, suggesting that MgAl2.0 improved the long-term thermal stability of PVC. Similarly, PVC with CaAl2.0, CaMgAl11, ZnMgAl11, and ZnAl2.0 changed from yellow to brown after 75, 80, 90, and 105 min of aging, respectively, indicating that the order of ZnAl2.0 = MgAl2.0 > ZnMgAl11 > CaMgAl11 > CaAl2.0 in terms of enhancing the long-term thermal stability of PVC. This order does not match the basic strength order of ZnAl2.0 > ZnMgAl11 > MgAl2.0 > CaMgAl11 > CaAl2.0 or the surface basicity order of MgAl2.0 > ZnAl2.0 > ZnMgAl11 > CaAl2.0 > CaMgAl11. This discrepancy could be due to the absorption of HCl by LDHs in the late stage of aging, leading to the formation of ZnCl_2_, MgCl_2_, and CaCl_2_, which have differing catalytic effects on PVC degradation in the order of ZnCl_2_ > CaCl_2_ > MgCl_2_. As reported by Ye et al. [10], the long-term thermal stability of PVC is also influenced by chloride species and their contents.

### 3.2. The Effect of Mg/Al Ratio in Laminae on the Surface Basicity of LDHs and the Thermal Stability of PVC Composites

**Figure 5:**
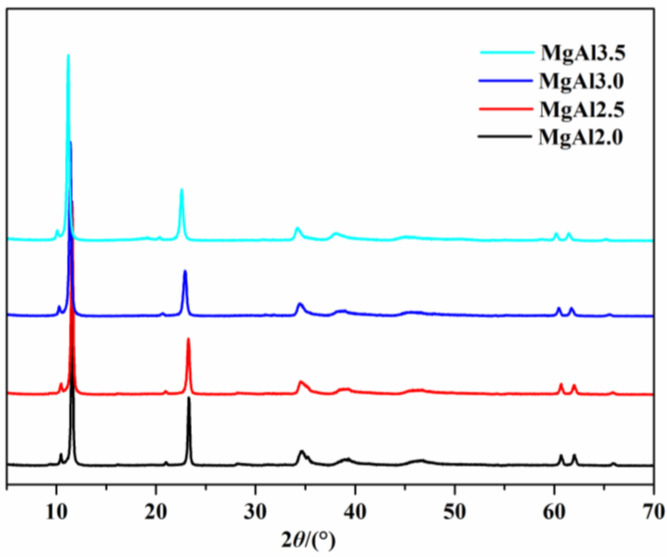
XRD patterns of LDHs with Mg/Al molar ratios of 2.0, 2.5, 3.0, and 3.5.

For the investigation of the relationship between the surface basic strength of hydroxyl groups and the surface basicity of LDHs, a series of Mg-Al LDHs were produced by varying the molar ratio of Mg/Al while keeping the metal elements in the laminae constant (Figure 5). All of the synthesized products displayed the characteristic diffraction peaks of hydrotalcite. As the molar ratio increased from 2.0 to 3.5, the d(003) characteristic diffraction peak of the LDHs gradually shifted to a smaller angle (2θ were 11.61°, 11.54°, 11.40°, and 11.25°, respectively), which is line with a previous literature report [27].

**Figure 6:**
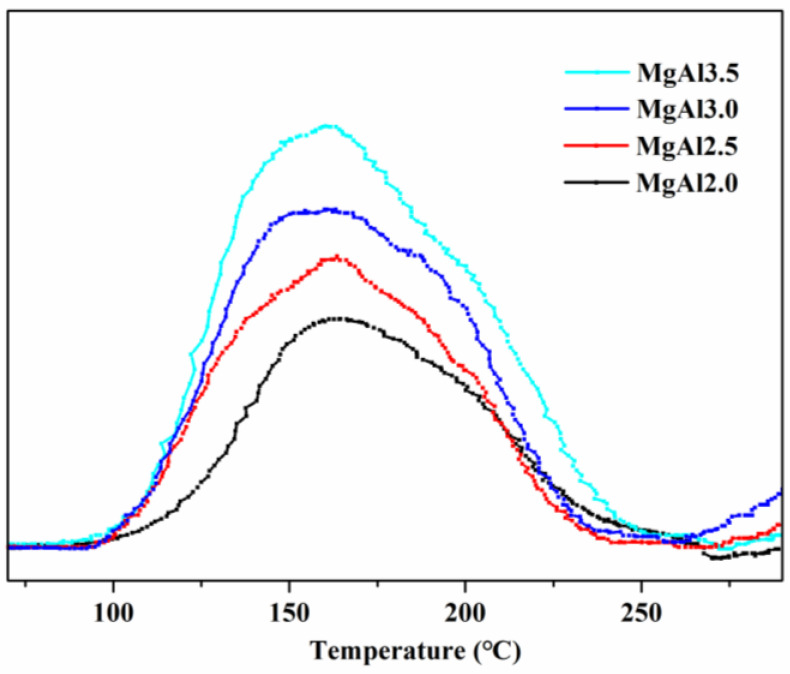
CO_2_-TPD profiles of LDHs with different Mg/Al molar ratios.

As shown in Figure 6 and Table 2, the desorption peak temperature decreased with the increase in the Mg/Al molar ratio from 2.0 to 3.5. In contrast, the peak area increased, indicating that while the strength of the basicity of LDHs declined, their surface basicity increased. The main hydroxyl groups of LDHs were Mg_3_OH and Mg_2_AlOH, with the former being higher in basicity than the latter. The proportions of Mg_3_OH were 3%, 20%, and 38% when the Mg/Al molar ratio was 2.0, 3.0, and 4.0, respectively [29]. This suggests that the content of Mg_3_OH in LDHs increased in the order of MgAl2.0 < MgAl2.5 < MgAl3.0 < MgAl3.5. This order agrees with the surface basicity order of LDHs (MgAl2.0 < MgAl2.5 < MgAl3.0 < MgAl3.5), implying that the surface basicity of LDHs is dependent on the Mg_3_OH groups.

**Figure 7:**
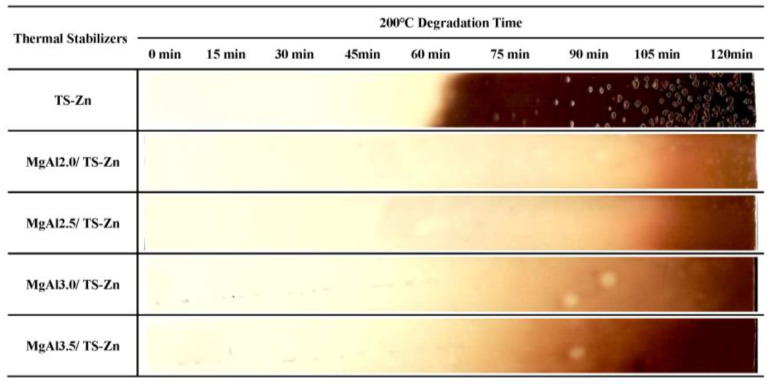
The molar ratio of Mg/Al in LDHs affects the thermal stability of PVC in an oven-aging test.

The thermal stability of PVC composites containing LDHs with different Mg/Al molar ratios was evaluated (Figure 7). The chromaticity index, b*, of PVC composites increased from 7.62 for MgAl2.0 to 8.95 for MgAl3.5 at 0 min aging, indicating that the composites became increasingly yellow as the Mg/Al ratio increased. This reduction in the initial whiteness of PVC composites was further evidenced by the decrease in the time required for the composites to turn yellow, from 90 min for MgAl2.0 to 60 min for MgAl3.5. The time needed for the composites to turn brown also decreased, from 105 min for MgAl2.0 to 75 min for MgAl3.5. These results suggest that as the Mg/Al molar ratio of LDHs increases, the long-term thermal stability of PVC composites deteriorates. This may be due to the increased Mg/Al ratio, making it easier for the PVC molecular chain to be attacked, releasing HCl and forming a conjugated polyene sequence, which reduces the stability and accelerates the consumption of thermal stabilizers. The initial whiteness and long-term thermal stability of PVC composites was dependent on the Mg/Al molar ratio of the LDHs, with smaller ratios resulting in whiter and more stable composites (MgAl2.0 > MgAl2.5 > MgAl3.0 > MgAl3.5). This order is consistent with the strength sequence of basicity (MgAl2.0 > MgAl2.5 > MgAl3.0 > MgAl3.5) but opposite to the surface basicity order (MgAl2.0 < MgAl2.5 < MgAl3.0 < MgAl3.5), meaning that increasing the strength of basicity and reducing the surface basicity of LDHs leads to whiter and more thermally stable PVC.

### 3.3. The Impact of Crosslinking on the Surface Basicity of LDHs and Thermal Stability of PVC Composites

**Figure 8:**
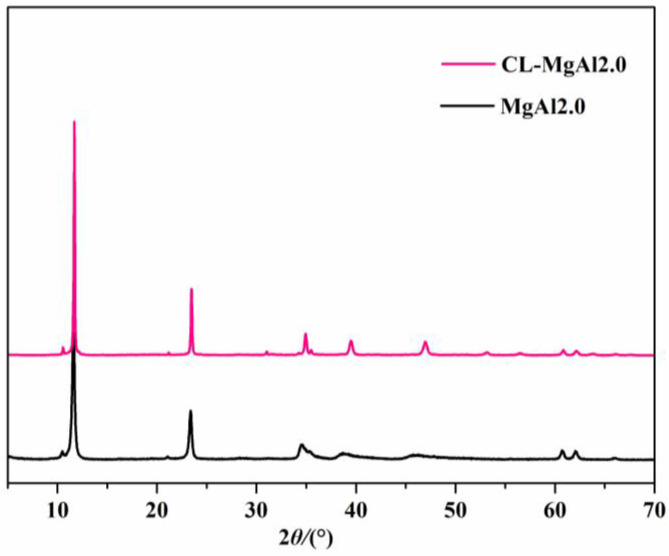
The XRD pattern of MgAl2.0 and its crosslinked counterpart (CL-MgAl2.0).

The surface basicity of LDHs is influenced not only by the types and contents of hydroxyl groups but also by crosslinking between the layers. To assess the relationship between the structure and surface basicity, the silane coupling agent KH560 was used to react with the hydroxyl groups on the layers while keeping the metal species and their ratios (Mg/Al = 2.0) constant. As shown in Figure 8, the crosslinked product (CL-MgAl2.0) also had the characteristic diffraction peaks of LDHs. Their positions were similar to MgAl2.0, indicating the interlayer spacings of both MgAl2.0 and CL-MgAl2.0 were consistent, and the layer charge density and interlayer anions were also the same. However, KH560 affected the surface of the LDHs.

**Figure 9:**
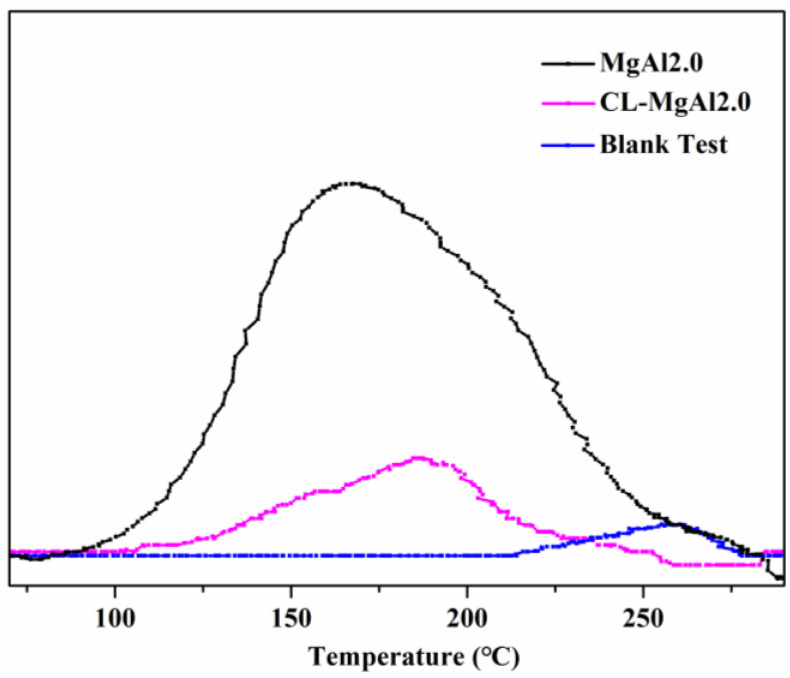
CO_2_-TPD diagrams of MgAl2.0 and its crosslinked counterpart (CL-MgAl2.0).

The desorption peak temperature of MgAl2.0 was 20°C lower than that of crosslinked CL-MgAl2.0, while the peak area of MgAl2.0 was much larger than that of its crosslinked counterpart (Figure 9 and Table 3). This suggests that crosslinking has increased the strength of the basicity and reduced the surface basicity of the LDHs.

**Figure 10:**
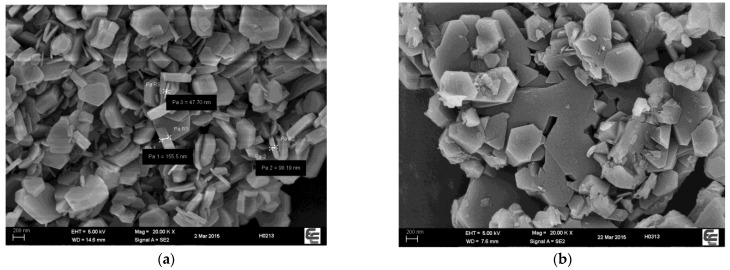
SEM images of MgAl2.0 (**a**) and its crosslinked counterpart CL-MgAl2.0 (**b**).

The SEM images of MgAl2.0 (Figure 10) depict randomly stacked laminae with distinct boundaries. Conversely, the laminae of crosslinked CL-MgAl2.0 were arranged in a more orderly manner, with “stitching” and “stacking” taking place among the laminae, leading to the formation of broader and thicker laminae, which indicates a clear crosslinking between the laminae and supports the results reported in previous works [35]. This may be due to the hydrolysis and condensation of some ethoxy groups of the silane coupling agent in the alkaline solution. The uncondensed -SiOH reacted with the more basic hydroxyl groups (Mg_3_OH) on the laminae of MgAl2.0, leading to a more stable and orderly stacking between the laminae. This hypothesis is supported by the BET-specific surface area measurements. MgAl2.0 had a specific surface area of 11.73 ± 0.04 m^2^/g, while the value was only 3.91 ± 0.18 m^2^/g for crosslinked CL-MgAl2.0. Overall, crosslinking had a similar effect to that of reducing the Mg/Al molar ratio in enhancing the basicity strength of LDHs and reducing their surface basicity.

**Figure 11:**
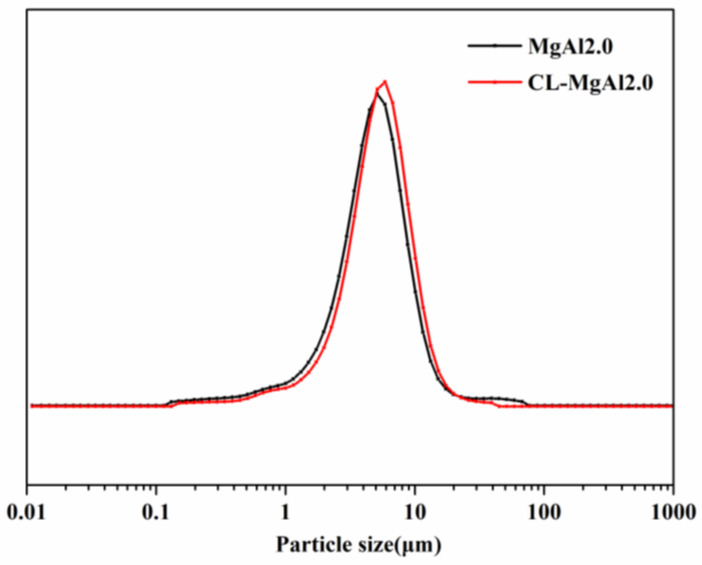
Particle size distribution (dry method) of MgAl2.0 and its crosslinked counterpart CL-MgAl2.0.

The particle size and size distribution of LDHs before and after crosslinking were measured using a laser particle size analyzer in the dry method, as shown in Figure 11 and Table 4. The particle size of both MgAl2.0 and CL-MgAl2.0 had a normal distribution, with most particles ranging from 2.0 to 9.5 μm. The average particle size (D0.5) was 4.51 μm for MgAl2.0 and 4.93 μm for CL-MgAl2.0. The results suggest that the particle size and size distribution of LDHs did not change significantly after crosslinking.

**Figure 12:**
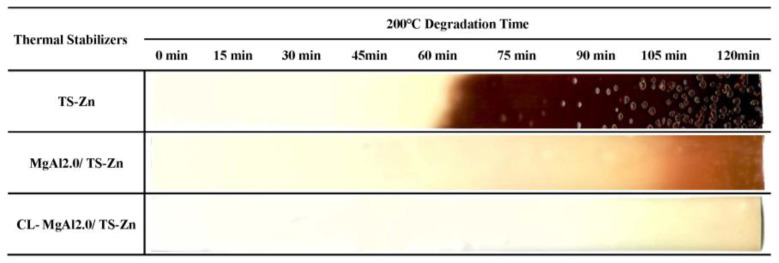
Comparison of the thermal stability of PVC reinforced with MgAl2.0 and its crosslinked counterpart CL-MgAl2.0.

The effects of crosslinking LDHs on the thermal stability of PVC composites are depicted in Figure 12. The composite containing MgAl2.0/TS-Zn had a chromaticity index, b*, of 7.62 after 0 min of aging. It turned yellow after 60 min of aging and became brown after 105 min. On the other hand, the composite with crosslinked MgAl2.0 (CL-MgAl2.0/TS-Zn) had a chromaticity index, b*, of 4.23 after 0 min, and it turned yellow and brown after 90 min and 120 min of aging, respectively. These results indicate that crosslinking improved the initial whiteness of the PVC composite and enhanced its long-term thermal stability by 14.3%. Combining the results from the CO_2_-TPD analysis (Figure 9 and Table 3), it is evident that crosslinking increased the strength of basicity, reduced surface basicity, improved initial whiteness, and enhanced the long-term thermal stability of PVC composites. The effect of crosslinking was similar to that of lowering the Mg/Al molar ratio in improving the initial whiteness and long-term thermal stability of PVC.

**Figure 13:**
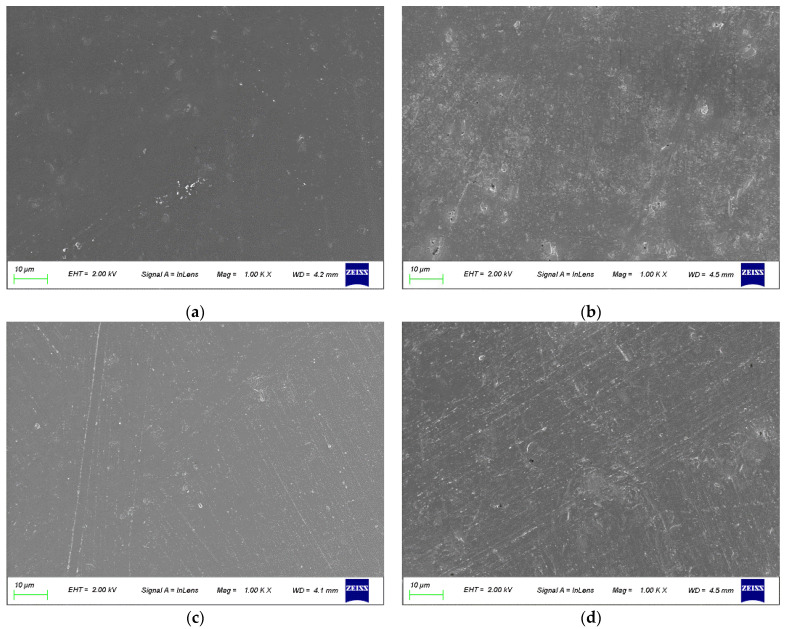
The SEM images of PVC composite materials at different aging times. MgAl2.0/TS–Zn (**a**—0 min; **b**—105 min) and CL-MgAl2.0/TS–Zn (**c**—0 min; **d**—105 min).

**Figure 14:**
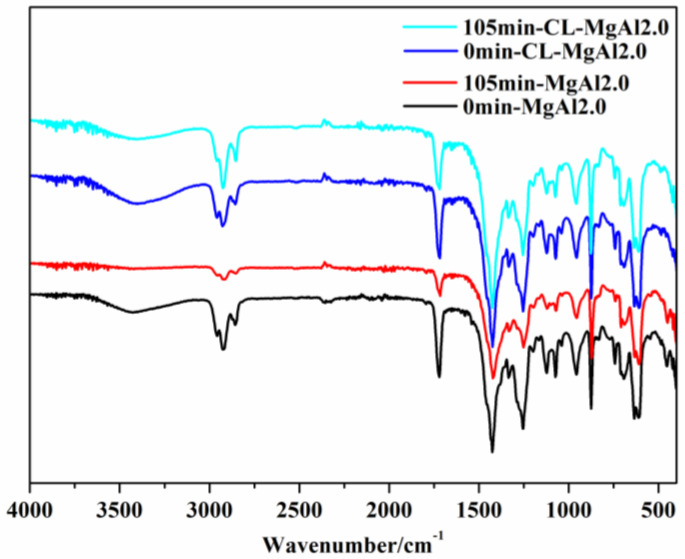
The ART-FTIR of PVC composites with MgAl2.0 and its crosslinked counterpart at aging times of 0 and 105 min.

To further investigate the impact of crosslinking on the thermal stability of PVC, SEM and ART-FTIR were performed on the PVC composite materials before and after aging. The outcomes are presented in Figure 13 and Figure 14. At 0 min of aging, the PVC composite materials with MgAl2.0/TS–Zn and CL-MgAl2.0/TS–Zn were smooth and compact, demonstrating successful PVC plasticization. After 105 min of aging, tiny pores emerged in the PVC composite materials containing MgAl2.0/TS–Zn and CL-MgAl2.0/TS–Zn, indicating that the PVC had decomposed. The PVC composite material containing MgAl2.0/TS–Zn showed larger pores, implying that its decomposition was more severe. This outcome aligns with the results of the oven aging process.

As shown in Figure 14, the ART-FTIR of PVC composite materials containing MgAl2.0/TS–Zn and CL-MgAl2.0/TS–Zn aged at different times have similar absorption peaks. The peaks at 1255, 694, and 616 cm^−1^ correspond to the C-Cl bonds in PVC, 1427 cm^−1^ is the -CH_2_- peak in PVC, 874 cm^−1^ is the benzene ring positional substitution peak in DOTP, 1719 cm^−1^ is the -C=O peak in DOTP, 1255 and 1123 cm^−1^ correspond to the -C-O in DOTP, 2959 and 2860 cm^−1^ are the -CH_3_ and -CH_2_- peaks, and 874 cm^−1^ is the characteristic peak of CaCO_3_. The -OH characteristic peak in LDHs is found between 3130 and 3670 cm^−1^.

After aging for 105 min, the 874 cm^−1^ CaCO_3_ peak remained unchanged, suggesting that the filler did not react. However, the intensity of the 1719 cm^−1^ -C=O peak decreased significantly due to the volatilization of DOTP during high-temperature aging. The C-Cl peaks in PVC, specifically those at 1255, 694, and 616 cm^−1^, also decreased significantly, with a more noticeable decrease in PVC composite materials containing MgAl2.0/TS–Zn, indicating that these materials underwent more severe decomposition and released more HCl. The -OH absorption peak in PVC composite materials containing MgAl2.0/TS–Zn nearly disappeared, while it was still visible in PVC composite materials containing CL-MgAl2.0/TS–Zn, which showed that MgAl2.0 was consumed more quickly, and the decomposition in PVC composite materials containing MgAl2.0/TS–Zn was more severe.

## 4. Conclusions

Developing environmentally friendly thermal stabilizers is critical for the successful application of PVC. In this study, LDHs with varying Mg/Al molar ratios and metal species were synthesized and cross-linked to evaluate their impact on PVC properties. The results showed that a lower Mg/Al ratio of 2.0 improved the initial whiteness and long-term thermal stability of PVC-LDH composites compared to higher ratios of 2.5, 3.0, and 3.5. Furthermore, substituting Mg in LDHs with Zn had a similar effect to that of lowering the Mg/Al molar ratio in improving PVC properties. A third approach, crosslinking the LDH laminae with 5% silane coupling agent KH-560, reduced the surface basicity by 79%, improved the initial whiteness of PVC by 48%, and increased its long-term thermal stability by 14%. These three approaches work by reducing the surface basicity of LDHs and enhancing their basic strength, reducing the negative effects of basicity on PVC molecular chains, such as the release of HCl, consumption of thermal stabilizer, and decreased stability of PVC. The weak basicity (<200 °C) of LDHs was primarily attributed to Mg_3_OH, while reducing the molar ratio of divalent and trivalent metal elements in LDHs and increasing the crosslinking degree of LDH layers significantly reduced their surface basicity. A medium to strong basicity (200–300 °C) of LDHs was caused by M-O (where M represents a metal element), and an increase in the content of metal elements with a high electron-donating ability strengthened the surface basicity of LDHs.

## 5. Patents

In relation to this research, a patent (ZL201910476975.4) was granted by China National Intellectual Property Administration.

## Figures and Tables

**Table 1:** Desorption peak temperature and area of LDHs with different metal elements in laminae.

LDHs	Temperature/°C	Area
MgAl2.0	165	1188
CaAl2.0	134	609
CaMgAl11	141	458
ZnAl2.0	225	982
ZnMgAl11	218	971

**Table 2:** Desorption peak temperature and area of LDHs with different Mg/Al molar ratios.

LDHs	Temperature/°C	Area
MgAl2.0	165	1188
MgAl2.5	158	1360
MgAl3.0	154	1718
MgAl3.5	153	2065

**Table 3:** Desorption peak temperature and area of MgAl2.0 and its crosslinked counterpart.

LDHs	Temperature/°C	Area
MgAl2.0	165	1188
CL-MgAl2.0	185	253

**Table 4:** Particle size (dry method) of MgAl2.0 and its crosslinked counterpart CL-MgAl2.0.

LDHs	D(0.1)/μm	D(0.35)/μm	D(0.5)/μm	D(0.75)/μm	D(0.9)/μm
MgAl2.0	1.69	3.59	4.51	6.48	9.13
CL-MgAl2.0	2.00	3.97	4.93	6.98	9.55

## Data Availability

Available upon request.

## References

[1] Avalos A.S., Hakkarainen M., Odelius K. (2017). Superiorly plasticized PVC/PBSA blends through crotonic and acrylic acid functionalization of PVC. Polymers.

[2] Abreu C.M.R., Fonseca A.C., Rocha N.M.P., Guthrie J.T., Coelho J.F.J. (2018). Poly(Vinyl Chloride): Current status and future perspectives via reversible deactivation radical polymerization methods. Prog. Polym. Sci..

[3] Tian W., Li Z., Zhang K., Ge Z. (2019). Facile synthesis of exfoliated vermiculite nanosheets as a thermal stabilizer in polyvinyl chloride resin. RSC Adv..

[4] Ye F., Ye Q., Zhan H., Ge Y., Ma X., Xu Y., Wang X. (2019). Synthesis and study of zinc orotate and its synergistic effect with commercial stabilizers for stabilizing poly(vinyl chloride). Polymers.

[5] Ye F., Guo X.J., Zhan H.H., Lin J.X., Lou W.C., Ma X.T., Wang X. (2018). The synergistic effect of zinc urate with calcium stearate and commercial assistant stabilizers for stabilizing poly(vinyl chloride). Polym. Degrad. Stab..

[6] Chen J., Liu Z., Wang K., Huang J., Li K., Nie X., Jiang J. (2018). Epoxidized castor oil–based diglycidyl–phthalate plasticizer: Synthesis and thermal stabilizing effects on poly(vinyl chloride). J. Appl. Polym. Sci..

[7] Wu B.Z., Wang Y.T., Chen S., Wang M.Y., Ma M., Shi Y.Q., Wang X. (2018). Bis–uracil based high efficient heat stabilizers used in super transparent soft poly (vinyl chloride). Polym. Degrad. Stab..

[8] Turner A., Filella M. (2021). Hazardous metal additives in plastics and their environmental impacts. Environ. Int..

[9] Shi Y., Yao Y., Lu S., Chen L., Chen S., He H., Ma M., Wang X. (2022). Synergistic effect of two plasticizers on thermal stability, transparency, and migration resistance of zinc arginine stabilized PVC. Polymers.

[10] Ye Q., Ma X., Li B., Jin Z., Xu Y., Fang C., Zhou X., Ge Y., Ye F. (2019). Development and investigation of lanthanum sulfadiazine with calcium stearate and epoxidised soyabean oil as complex thermal stabilizers for stabilizing poly(vinyl chloride). Polymers.

[11] Jubsilp C., Asawakosinchai A., Mora P., Saramas D., Rimdusit S. (2022). Effects of organic based heat stabilizer on properties of polyvinyl chloride for pipe applications: A comparative study with Pb and CaZn systems. Polymers.

[12] Li Y., Li D., Han W., Zhang M., Ai B., Zhang L., Sun H., Cui Z. (2019). Facile Synthesis of di–mannitol adipate ester–based zinc metal alkoxide as a bi–functional additive for poly(vinyl chloride). Polymers.

[13] Li D., Xie L., Ming F., Zhang J., Indrawirawan S., Zhang Y. (2015). Synergistic effects of lanthanum–pentaerythritol alkoxide with zincstearates and with β–diketone on the thermal stability of poly(vinyl chloride). Polym. Degrad. Stab..

[14] Guo Y., Leroux F., Tian W., Li D., Tang P., Feng Y. (2021). Layered double hydroxides as thermal stabilizers for poly(vinyl chloride): A review. Appl. Clay Sci..

[15] Ven L.V.D., Gemert M.L.M.V., Batenburg L.F. (2000). On the action of hydrotalcite–like clay materials as stabilizers in polyvinylchloride. Appl. Clay Sci..

[16] Chen Y., Zhang S., Han X., Zhang X., Yi M., Yang S., Yu D., Liu W. (2018). Catalytic dechlorination and charring reaction of polyvinyl chloride by CuAl layered double hydroxide. Energy Fuel.

[17] Labuschagne F., Dan M.M., Focke W.W., Westhuizen I. (2015). Heat stabilising flexible pvc with layered double hydroxide derivatives. Polym. Degrad. Stab..

[18] Wen R., Yang Z., Chen H., Youwang H.U., Duan J. (2012). Zn–al–la hydrotalcite–like compounds as heating stabilizer in pvc resin. J. Rare Earths.

[19] Yan J., Yang Z. (2017). Intercalated hydrotalcite-like materials and their application as thermal stabilizers in poly(vinyl chloride). J. Appl. Polym. Sci..

[20] Liu S.T., Zhang P.P., Yan K.K., Zhang Y.H., Ye Y., Chen X.G. (2015). Sb–intercalated layered double hydroxides–poly(vinyl chloride) nanocomposites: Preparation, characterization, and thermal stability. J. Appl. Polym. Sci..

[21] Zhang X., Zhao T., Pi H., Guo S. (2012). Preparation of intercalated Mg–Al layered double hydroxides and its application in pvc thermal stability. J. Appl. Polym. Sci..

[22] Gao Z., Lu L., Shi C., Qian X.-D. (2020). The effect of OCoAl–LDH and OCoFe–LDH on the combustion behaviors of polyvinyl chloride. Polym. Adv. Technol..

[23] Yang H., Yang Z. (2017). The effect of sodium stearate-modified hydrocalumite on the thermal stability of poly(vinyl chloride). J. Appl. Polym. Sci..

[24] Zhang H.M., Zhang S.H., Stewart P., Zhu C.H., Liu W.J., Hexemer A. (2016). Thermal stability and thermal aging of poly(vinyl chloride)/mgal layered double hydroxides composites. Chin. J. Polym. Sci..

[25] Wen X., Yang Z.H., Yan J., Xie X. (2015). Green preparation and characterization of a novel heat stabilizer for poly(vinyl chloride)–hydrocalumites. RSC Adv..

[26] Mori K., Miyata S. (2019). Particulate Hydrotalcite, Its Manufacturing Method, Its Resin Composition, and Suspension Thereof. Patent Application.

[27] Lin Y.J., Li D.Q., Evans D.G., Xue D. (2005). Modulating effect of Mg–Al–CO_3_ layered double hydroxides on the thermal stability of pvc resin. Polym. Degrad. Stab..

[28] Jia L., Yin L., Luo Z., Wang H., Zhong W. (2016). Molecular chain model construction, thermo–stability, and thermo–oxidative degradation mechanism of poly (vinyl chloride). RSC Adv..

[29] Sideris P.J., Nielsen U.G., Gan Z., Grey C.P. (2008). Mg/Al ordering in layered double hydroxides revealed by multinuclear NMR spectroscopy. Science.

[30] Yu G., Zhou Y., Yang R. (2015). Dehydration and dehydroxylation of layered double hydroxides: New insights from solid–state NMR and FT–IR studies of deuterated samples. J. Phys. Chem. C.

[31] (2019). Determination of Thermal Stability of Poly(Vinyl Chloride) Related Chlorine–Containing Homopolymers and Copolymers and Their Compounds––Discoloration Method.

[32] Monica L.J., Siti H.S., Zaemah J. (2022). Synthesis and characterisation of layered double hydroxides with varying divalent metal cations: Mg^2+^, Zn^2+^, Ca^2+^. Mater. Today.

[33] Yan R.D., Oscar W.P.L. (2023). CO_2_ methanation over Ni–Al LDH–derived catalyst with variable Ni/Al ratio. J. CO_2_ Util..

[34] Tomaszewska J., Sterzynski T., Walczak D. (2021). Thermal stability of nanosilica–modified poly(vinyl chloride). Polymers.

[35] Tao Q., Zhu J., Wellard R.M., Bostrom T.E., Frost R.L., Yuan P., He H. (2011). Silylation of layered double hydroxides via an induced hydrolysis method. J. Mater. Chem..

